# Towards Physiologic Culture Approaches to Improve Standard Cultivation of Mesenchymal Stem Cells

**DOI:** 10.3390/cells10040886

**Published:** 2021-04-13

**Authors:** Ilias Nikolits, Sabrina Nebel, Dominik Egger, Sebastian Kreß, Cornelia Kasper

**Affiliations:** Institute for Cell and Tissue Culture Technology, Department of Biotechnology, University of Natural Resources and Life Sciences, Muthgasse 18, 1190 Vienna, Austria; ilias.nikolits@boku.ac.at (I.N.); sabrina.nebel@boku.ac.at (S.N.); sebastian.kress@boku.ac.at (S.K.); cornelia.kasper@boku.ac.at (C.K.)

**Keywords:** mesenchymal stem cells, physiologic cultivation, optimization cultivation

## Abstract

Mesenchymal stem cells (MSCs) are of great interest for their use in cell-based therapies due to their multipotent differentiation and immunomodulatory capacities. In consequence of limited numbers following their isolation from the donor tissue, MSCs require extensive expansion performed in traditional 2D cell culture setups to reach adequate amounts for therapeutic use. However, prolonged culture of MSCs in vitro has been shown to decrease their differentiation potential and alter their immunomodulatory properties. For that reason, preservation of these physiological characteristics of MSCs throughout their in vitro culture is essential for improving the efficiency of therapeutic and in vitro modeling applications. With this objective in mind, many studies already investigated certain parameters for enhancing current standard MSC culture protocols with regard to the effects of specific culture media components or culture conditions. Although there is a lot of diversity in the final therapeutic uses of the cells, the primary stage of standard isolation and expansion is imperative. Therefore, we want to review on approaches for optimizing standard MSC culture protocols during this essential primary step of in vitro expansion. The reviewed studies investigate and suggest improvements focused on culture media components (amino acids, ascorbic acid, glucose level, growth factors, lipids, platelet lysate, trace elements, serum, and xenogeneic components) as well as culture conditions and processes (hypoxia, cell seeding, and dissociation during passaging), in order to preserve the MSC phenotype and functionality during the primary phase of in vitro culture.

## 1. Introduction

Cell-based therapies aim to repair or regenerate defective tissues or organs due to physical or congenital damage, ageing, and degenerative diseases. From a regulatory viewpoint cell-based therapies can be divided between minimally manipulated cells for homologous use like transplants or transfusions and somatic cell, gene therapy, and tissue engineered products, which are referred to as Advanced Therapy Medicinal Products (ATMPs) by EU authorities [1]. These are considered medicines and need to comply to quality, safety, and efficacy standards before getting a market authorization. Generally, when referring to cell-based therapies, not only cells as medicinal product, but also products derived from them are included [2]. 

One possible treatment approach in cell-based therapies is the engraftment of cells into the damaged site to replace or restore the defective cells or tissue regarded as classical cell therapy. Another mechanism is based on the stimulating effect of trophic factors, through targeted drug delivery or cell genetic manipulation, to promote endogenous self-regeneration of the injured tissue. Cell-based therapies are a very promising strategy for the treatment of many severe and until recently considered incurable vascular, neurological, autoimmune, ophthalmologic, and skeletal diseases [3]. 

Among the different cell types tested for their therapeutic potential, stem cells are the most prominent, considering their intrinsic self-renewal and differentiation capacities [4]. Particularly, mesenchymal stem cells (MSCs) are of great interest for their use in cell-based therapies. MSCs are highly proliferative multipotent stromal cells that can differentiate into the mesodermal cell lineage [5]. Due to the therapeutic potential of MSCs, many studies focus on their characterization and identification. According to the International Society for Cellular Therapy (ISCT), MSCs must be plastic-adherent and differentiate into osteoblasts, chondroblasts, and adipocytes when cultured in vitro. In addition, they should express CD90, CD73, and CD105 surface markers and should not express CD34, CD45, CD14 or CD11b, CD19 or CD79A and HLA-DR surface molecules [6]. MSCs can be derived from various tissue sources such as the bone marrow [7], adipose tissue [8], umbilical cord [9], peripheral blood [10], and dental pulp [11], but the number of primary MSCs isolated from a donor tissue is rather limited [5] and source-dependent [12]. 

With the introduction of cell culture techniques, traditional two-dimensional (2D) cell culture has been for decades the backbone method for medical and biological research and drug development due to its simplicity and robustness. However, the static and planar conditions of 2D culture fail to simulate the physiological environment of the cells. For that reason, during the last years, concerns over the biological relevance and reproducibility of in vitro culture techniques have led to the development of advanced three-dimensional (3D) and dynamic cell culture methods. These methods serve as better models for the representation of in vivo physiological conditions [13,14], as well as platforms for up-scaling production of cells for clinical applications [15,16]. As a result, several 3D cell culture techniques now exist, from multicellular spheroids/organoids [17,18] and cell-laden biomaterials [19,20,21,22], to dynamic bioreactor systems [23,24,25,26] and organ/body on a chip systems [27,28,29]. It has been shown that such 3D culture systems preserve many characteristics and properties that resembled MSC behavior and differentiation in vivo [30,31,32]. Therefore, the gained results are more relevant for the translation into clinical applicability. However, these 3D advanced cell cultures require high numbers of cells [16], are more complex to establish and maintain, and are therefore more laborious.

Due to the fact that MSCs are a very limited adult tissue population [33,34], following their isolation from donor tissue, harvested primary MSCs should be expanded first in 2D culture setups, to reach adequate numbers for use in various in vitro modelling and therapeutic applications [5]. However, prolonged culture of MSCs in vitro in 2D systems has shown to decrease their differentiation potential [35,36] and alter their phenotype [37] and immunomodulatory properties [38]. In order to improve the relevance of in vitro modelling and efficiency of therapeutic applications, it is essential to preserve the physiological properties and characteristics of MSCs throughout their in vitro culture. 

In view of this, there is a considerable research focus on the optimization of culture conditions and protocols for MSCs. Since there is yet no medium that is as close to natural in vivo fluids to support physiological MSC culture, specialized culture media are composed based on the cell type, type of culture, and focus of analysis. Additional focus is put on the effects of other cell culture variables, including physiochemical conditions and subculture protocols. As a result, between different cell culture laboratories, there is divergence on these culture variables. Therefore, it is necessary to define what constitutes an “optimal practice” for maintaining the physiological status of MSCs, during their primary step of in vitro 2D expansion. Further, during optimization of cultivation procedures considerations on other markers of success next to population growth should be considered to circumvent potentially highly proliferative, but severely altered cell populations. In a recent position statement, the ISCT underlines that MSCs often lose their functional properties during in vitro expansion, and it is suggested to verify relevant functionalities of MSCs by applying a matrix of functional assays based on the later therapeutic use [39]. Therefore, we want to review on optimizing approaches for standard 2D in vitro expansion of human MSCs, focusing on culture media components (amino acids, ascorbic acid, glucose level, growth factors, lipids and fatty acids, platelet lysate, trace elements, serum, and xenogeneic components) as well as culture conditions and processes (hypoxia, seeding density and cell dissociation during passaging).

## 2. Basal Media for Isolation and Expansion

In vitro culture of MSCs is performed using a defined basal medium. Several basal media with different formulations are commercially available for culture of human MSCs, and the choice of basal media used varies between different laboratories. These media primarily contain glucose, amino acids, and trace elements which are necessary for cellular growth and survival. Regarding the selection of basal medium for optimal expansion MSCs, Sotiropoulou et al., cultured primary bone marrow mononuclear cells and passaged bone marrow MSCs (BM-MSCs) in the presence of different media (see Table 1). According to their results, cultures of primary and passaged MSCs in aMEM/GL and aMEM/L-G media generated the highest number of cells, with minor differences in their phenotype over subsequent passages [40]. In another study comparing 4 different basal media, adipose tissue MSCs showed increased proliferation when aMEM, but also DMEM/LG, were used as basal media, without changes in their morphology and viability during subsequent passages [41]. In a similar study comparing different culture media, a variation of “Verfaillie” medium with DMEM/HG/L-G yielded higher number of adherent MSCs from primary mononuclear bone marrow cell populations and higher proliferation rate of BM-MSCs at early passages, compared to other media formulations, including aMEM/L-G and DMEM/LG/L-G based media [42]. In addition, studies comparing different types of media with BM-MSCs reported significant changes in their phenotypic expression profile [42,43], in contrast with a study using adipose-derived MSCs where different basal media did not alter the MSC specific marker expression after multiple passages [41]. As indicated by the literature, the optimal choice of basal media can vary, depending on the type and of MSCs cultured, although in general, aMEM based media have been indicated as the most suitable for isolation and expansion of MSCs. Furthermore, evaluation of the optimal choice of basal media should not only depend on its effect on the proliferative capacity of MSCs, but also on the preservation of their intrinsic characteristics. Researches should test and consider both aspects regarding their choice of basal media for their MSC cultures.

## 3. Glucose

Glucose is a basic source of energy for the cells and is involved in the synthesis of proteins and lipids. In vivo, plasma glucose concentration for healthy people ranges between 700–1000 mg/L throughout the day, and it does not exceed 1600 mg/L after meals. Glucose plays an essential role in survival, metabolism, and physiological function of MSCs. In cell culture media formulations glucose concentration ranges from 1000 mg/L to resemble physiological in vivo conditions, up to 10,000 mg/L. Culture media formulations with glucose concentrations higher than 1000 mg/L simulate in vivo diabetic conditions [44]. Due to the highly glycolytic phenotype of MSCs, glucose is more rapidly consumed and its depletion has stronger effects on the cells compared to other nutrients and serum [45]. According to the literature however, the effects of the amount glucose on MSC cultures are diverse, depending on the metabolic activity and the type of MSCs. Most studies under normoxic culture conditions support that high glucose concentration (5000 mg/L) in the culture media can suppress proliferation of bone marrow [46,47,48], Bichat’s buccal fat pad [49] and nucleus pulposus MSCs [50], reduce their colony forming ability [50], induce cellular senescence [47,50,51], alter MSC spindle-shape morphology [47], and upregulate autophagy [50,51], compared with low glucose (1000 mg/L) media concentration. In addition, MSCs cultured in low glucose media have been shown to maintain their differentiation capacities and stemness [46,47,50], and increase antioxidant enzyme expression and mitochondrial respiration [47]. In contrast with these results, a few studies have reported that high glucose does not have an effect on the apoptosis and proliferation rates of adipose-derived [52] and primary BM-MSCs [53], while it can significantly enhance proliferation of telomerase-immortalized MSCs. While it is known that under hypoxic culture conditions the metabolic activity of MSCs is enhanced [54], Deschepper et al., observed that under continuous hypoxic culture, excess of glucose provided by high glucose concentration media prevents glucose shortage during culture, thus preserving the proliferative capacity, morphology, and viability of BM-MSCs [55].

## 4. Amino Acids

Already in the first days of cell culture, it became clear that for cultivation ex vivo essential nutrients have to be supplied to cells, one of the most prominent categories being amino acids. Well known in the field of cell culture are Dulbecco and Eagle, who pioneered basal medium for cell culture that is still in use today, known as DMEM (Dulbecco’s modified eagle’s medium), usually supplemented with fetal bovine serum (FBS) [56]. With the aspiration to get rid of ill-defined xenogeneic supplements and to transition to chemically defined media, understanding and optimizing amino acid formulations is becoming even more important. In the past amino acid concentrations in culture medium have been chosen according to the consumption by the cells. However, degree of availability of an amino acid can have influence on the cellular metabolism therefore refined studies on their cellular metabolic properties are called for. Other parameters like solubility, stability, and transport into the cells also determine availability and further consumption of amino acids [57]. In the last years, it also became obvious that different mammalian cell types have completely different amino acid requirements and that medium optimization in terms of amino acid metabolism have to be fitted to the individual culture [58,59].

Of the 20 naturally occurring amino acids l-Arginine, l-Cysteine, l-Glutamine, l-Histidine, l-Isoleucine, l-Leucine, l-Lysine, l-Methionine, l-Phenylalanine, l-Threonine, l-Tryptophan, l-Tyrosine, and l-Valine are regarded as essential and Glycine, l-Alanine l-Asparagine, l-Aspartic, l-Glutamic, l-Proline, and l-Serine as non-essential, thus generally supplied with the basal media are the essential ones, as non-essential ones can be synthesized by the cells [60]. In a 2007 paper by Choi et al. [61], six different medium compositions with essential amino acids (EAAs) and non-essential amino-acids (NEAAs) were tested for their efficacy to promote proliferation of BM-MSCs. Their basal media were DMEM (basically containing EAAs) and IMDM (Iscove’s modified Dulbecco’s Medium) (basically containing EAAs and NEAAs), where either essential or non-essential amino acids or both were additionally added. They could see that oversupply with essential AAs as well as supplementation with non-essential AAs improved proliferation, whereas oversupply of NEAAs resulted in lowered proliferation. In none of the medium conditions did a change in stemness markers occur. Although this study did not perform design of experiment to tune each amino acid concentration individually it is one step in the direction of establishing improved culture conditions. Another approach to determine the nutritional requirements of MSCs in particular is the analysis of metabolic patterns, in detail which and in what concentration are amino acids consumed or secreted. They also tested whether this was consistent in 2D monolayer culture compared to dynamic culture on microcarriers. EAAs were consumed in both set ups as expected, but NEAAs were differently metabolized. MSCs consumed cysteine and proline in dynamic culture, but secreted them in static culture. However, results of static and dynamic agree on alanine, glutamate, glycine, and ornithine being produced, and arginine, asparagine, aspartate, glutamine, serine, and tyrosine being consumed by the cells. This consumption was not seen in other cell types before and might be unique to stem cells [62]. The difference of amino acid metabolism from static to dynamic culture indicates that the mechanisms are even more complex than thought, and optimizing medium composition is also influenced by cultivation techniques. Regarding one specific amino acid, already Eagle, et al. [63] describe the importance of glutamine for mammalian cell proliferation. Specifically for BM-MSCs, both dos Santos, et al. [64] and Zhou, et al. [65] report improved proliferation and very importantly maintenance of stemness. Additionally, oversupply of branched chain AAs (BCAAs), valine, leucine, and isoleucine increased S/G2/M cell cycle phases of a murine MSC lineage and could also improve the immunomodulatory capacity of the cells [66]. Although the minimal supply of amino acids is provided by the basal medium, these results show oversupply of certain amino acids can improve MSC proliferation, stemness, or immunomodulatory effects. The choice of AAs to be supplemented has to be made according to the intended application of the cells as well as the culture method. 

## 5. Lipids

Lipids are next to amino acids, sugars, and nucleic acids as one of the major classes of biomolecules that play important roles in the cell’s membrane structure, metabolism, and signaling. During the last decades, it has become obvious that disturbances in the lipid homeostasis are involved in the development of diseases like diabetes, cardiovascular disorders, autoimmune diseases, and even cancer development [67]. Not only understanding the lipidomics in vivo is of utter importance, but also the mechanisms of lipid metabolism in vitro are important to improve cell-based treatment strategies of those disorders. The most common forms of lipids in the cell are phosphatidylcholine (PC), phosphatidylserine (PS), phosphatidylinositol (PI), phosphatidylethanolamine (PE) [68], and cholesterol, which make up the outer cell membrane and the membrane of different organelles [60]. Next to this, triglycerides serve as energy storage within reservoirs (lipid droplets). The triglycerides can be broken down to fatty acids and used to produce ATP if needed. Lipids can also bind to proteins, making them useable as signaling molecules for extra- or intracellular messaging [60,69]. Almost all lipids necessary for basic cellular functions in mammalian cells can be produced by themselves, only two are regarded as “essential”: Linoleic and linolenic acid. However, in a lot of cases, even media without them allowed cells to proliferate, whereas addition drastically improved the results, suggesting differentiation between minimal requirement and optimal performance [70]. Requirements needed for substantial cellular proliferation, were traditionally met by the excess of lipids in the supplemented FBS. With the increasing interest to omit animal products (discussed in detail in “human platelet lysate” and “Serum-, xeno-free- and chemically defined media”), other delivery strategies have to be found. The hydrophobic nature of lipids needs different strategies to disperse lipids, allow cellular uptake, and remain stable in the medium. Three approaches have been described: (1) Adsorption to a soluble carrier molecule, (2) self-assembly to the required size, and (3) dispersion [71]. The mechanism in FBS is the adsorption to the serum proteins, especially to the bovine serum albumin (BSA), a strategy that can be used also in chemically defined media that are supplemented with some form of serum albumin (human or bovine, natural or recombinant origin). Dispersion techniques include liposomes, emulsion, and microemulsions and have the advantage that they can be used in animal component free and even protein free medium (see “Serum-, xeno-free- and chemically defined media”). These requirements are true for mammalian cell lines in general, yet better understanding of the exact lipidomics especially within stem cells is necessary to understand possible alterations during long term ex vivo maintenance and lead to establishment of improved cultivation environments [72]. For example, in a 2017 paper by Chatgilialoglu et al., they showed that the fatty acid composition of the membrane of primary human fetal membrane-derived MSCs changed in 2D culture over time [73]. The amount of omega-6 fatty acids decreased, whereas monounsaturated fatty acids (MUFA) and omega-3 fatty acids increased. Additionally, Kilpinen et al., showed changes in membrane composition of BM-MSCs [74]. In order to decrease the effect of prolonged culture on the cell membranes, Chatgilialoglu et al., developed a cell medium supplement. Refeed^®^ supplement is a completely defined combination of lipids and lipophilic antioxidants in ethanol. The supplementation was able to increase cellular proliferation compared to the control, without changes in surface marker composition [73]. With the advances in the field of omics, more complex interactions can be monitored. For example, differences in whole lipidomics of early and late passage cells were investigated using ultraperformance liquid chromatography coupled to mass spectrometry (UPLC–MS) and multivariate analysis. Global analysis revealed that changes in lipid significance and alterations during BM-MSC aging could be mostly contributed to chain length alterations and level of unsaturation [75]. An important aspect to consider was taken up by Fillmore et al. [76], who cultured BM-MSCs in physiological levels of fatty acids and serum albumin, which are higher than in standard cultivation conditions, to have a look on energy metabolism and survival. They could show that the addition of physiological levels of palmitate, a saturated fatty acid, significantly decreased BM-MSC viability in a time and concentration dependent manner. They could rescue this decrease in survival with the addition of equal amounts of oleate, an unsaturated fatty acid. By investigation of the metabolic activities, they conclude that decreasing fatty acid oxidation may be the source of cell death. These results are important to understand limited efficacy of cell-based therapies at the moment. Decreasing the amount of circulating saturated fatty acids in the patient during treatment might overcome current limitations in cell homing and survival after implantation [76]. With novel lipidomic tools, like, e.g., tandem MS-based, high mass accuracy-based, or multi-dimensional MS-based shotgun lipidomics [77] and improved analysis software [78,79], more insight is gained into the impact of lipids and fatty acids on MSCs ex vivo, which is the first step towards finding solutions to prevent unwanted alterations. As a general rule, media supplemented with FBS grants the cells a sufficient fatty acid supply, whereas human platelet lysate supplemented media might (depending on the manufacturing method) and serum/xeno-free medium need ancillary supply with lipids. Available are enriched forms or extracts from natural sources like palm oil, wool, serum, or fish [80], however for xeno-free culture synthetic alternatives to animal-derived supplements need to be used. 

## 6. Growth Factors

Over the last decades, different growth factors have been widely studied for their role of improving ex vivo expansion of human MSCs. Due to the pleiotropic behavior of these factors, they can affect multiple biological behaviors like proliferation, morphology, immunophenotype, survival, and differentiation capacity of MSCs. Selection of which growth factor or combination of factors can promote physiological culture of MSCs in vitro, is still under investigation. Many different growth factors have been investigated for their potential effects on expanding MSCs. From them, fibroblast growth factors-2 and -4 (FGF-2, FGF-4), platelet derived growth factor-BB (PDGF-BB), and epidermal growth factor (EGF) have been reported to influence human MSC proliferation and survival in vitro [81].

### 6.1. Fibroblast Growth Factor-2 and -4 

FGFs is involved in tissue repair and cell growth. FGF-2 used in many culture studies can enhance the proliferative [40,82,83,84,85,86,87] and colony-forming capacity [88] of MSCs, suppress cellular senescence [87,89], reduce trypsinization time [84], and preserve the thin spindle-shape morphology of MSCs [84,90]. In a study by Battula et al., primary bone marrow and placental MSCs cultured in gelatin-coated flasks with serum-free medium supplemented with FGF-2 demonstrated significantly higher proliferation rates than control cultures with serum containing media in uncoated flasks. Combination of FGF-2 with platelet derived growth factor-BB (PDGF) and/or transforming growth factor-β (TGF-β) in the culture media was also reported to increase the proliferation of human MSCs [83,88,90] and preserve the spindle-shaped morphology of MSCs in serum-free media conditions [90]. However, some studies have reported that FGF-2 supplement in human MSC cultures can affect the phenotype and intrinsic properties of the cells [40,83]. FGF-4, similarly to FGF-2, has been reported to increase proliferation, reduce autophagy, and delay the decrease of cellular renewal capacity of human MSCs [87].

### 6.2. Platelet Derived Growth Factor-BB 

PDGF-BB is a known regulatory factor of cellular growth and division. It has been noted that when PDGF-BB is supplement alone [88,91] or in combination with other factors [84,88,90] in the culture media, it can highly increase human MSC proliferation, while inhibition of its receptor can significantly impair their proliferative capacity [92]. Regardless, reports have indicated that it can also decrease colony forming efficiency [88] and osteogenic differentiation potential of cultured MSCs [91].

### 6.3. Epidermal Growth Factor

EGF and Heparin-Binding Epidermal Growth Factor (HB-EGF) have been shown to play a role in promoting wound healing and cell growth. Human BM-MSC cultures supplemented with EGF have demonstrated increased proliferation similar to the effect of PDGF stimulated proliferation [88,93], but without diminishing their osteogenic potential [91,93]. Krampera et al., have demonstrated that BM-MSCs cultured with HB-EGF can proliferate more rapidly without undergoing spontaneous differentiation, thus maintaining their multilineage differentiation potential during culture [94].

In the study by Fraz et al., vascular endothelial growth factor (VEGF), and insulin-like growth factor (IGF) in combination with FGF-2 were reported to improve MSC proliferation. More interestingly, from the same study it was reported that high initial growth factors contents in the MSC population, or pre-incubation of the cells with growth factors, can significantly diminish their impact on the proliferative capacity of MSCs when added as supplements in the culture medium [82].

Overall, the growth factors discussed above have been shown to be beneficial for MSC expansion when added to the medium and consist considerable components for standard culture media formulations. However, most studies have examined the effects of these factors when added solely to the media. That gives room to further investigation and analysis regarding the exerted effect from the combination of these growth factors during MSC culture.

## 7. Trace Elements

Culture media apart from the organic substances, also contain inorganic elements such as Cu, Mg, Mn, Zn, Se, Ca, and Fe in trace quantities. Serum used in cell cultures usually contains many of these elements, and therefore many basal media do not include them in the formulations. These trace elements can influence important biochemical functions of the cells, but their effect and exact biological role on MSCs have not been thoroughly investigated.

Zinc is involved with nucleic acid metabolizing enzymes and stabilization of cell membranes. Serum used usually in cell cultures contain the necessary amounts of zinc for cell growth and survival. For that reason, many basal media do not contain zinc. Addition of ZnCl_2_ in adipose tissue derived MSCs culture has been shown to highly enhance their proliferation, while zinc chelation reversed this effect. The exact mechanism of this effect has not been fully elucidated yet [95].

Magnesium acts as a cofactor for many enzymatic processes and as a counter ion for nucleic acids and ATP [96]. Human MSCs cultured with magnesium-conditioned medium have demonstrated increased viability and proliferation. Furthermore, magnesium-conditioned medium with Zinc and/or Manganese additions were shown to further promote human MSC proliferation and viability in the same study [97].

Calcium is an important element for a variety of biological functions including cell attachment, signaling, and morphology. Studies have shown that increased concentrations of extracellular calcium ions can significantly promote proliferation of BM-MSCs cultured in serum [98] and serum-free [99] media conditions, compared to controls. Increased calcium supplementation in the culture medium can also induce morphological changes on MSCs, resulting in more spread out cytoskeleton arrangements, which could be an indication towards their differentiation commitment [98].

Iron plays an essential role in cell metabolism and respiration. It protects cells from oxidative damage acting as an enzyme cofactor, but it can also cause toxic effects. Its delivery to the cells is controlled by transferrin, included in serum. That is why serum-free media formulations have a higher risk to cause iron toxic effects to cells. Borriello et al. have demonstrated that iron supplementation to the culture medium of primary human BM-MSCs, can greatly enhance their proliferation and initiate their S phase. Iron addition also hindered the innate osteogenic commitment of BM-MSCs, by blocking matrix calcification and preserving their undifferentiated status [100]. 

Selenium is included in enzymes that prevent oxidative damage to the cells by reducing peroxides and other oxidative free radicals to non-harmful forms. In cell culture media is usually added as selenium, sodium selenite, or selenium dioxide. The literature results on the effects of selenium supplementation on human MSCs are quite diverse. Selenium can restore the low antioxidative capacity of primary human BM-MSCs, and prevent cellular damage [101]. Park et al., found out that selenium can enhance the proliferation of human amniotic fluid MSCs, while combination of selenium with FGF-2 supplementation exerts an additive effect on the proliferative capacity of the cells [102]. Conversely, other studies have reported that selenium reduces the colony-forming ability of human MSCs [88] and does not have any significant effect on their doubling time [101].

Copper is important for cell growth and development, notably of the skeleton by affecting bone turnover and metabolism. Addition of copper in different concentrations in human BM-MSC cultures has been reported to decrease their proliferation in a dose dependent manner [103]. Since increased copper concentrations did not affect cell viability or cell size to justify contact inhibition of proliferating cells, it was hypothesized that copper could interfere with multiple cell cycle stages to hamper MSC proliferation [103].

Although studies have shown that trace elements included in the media can affect the biological behavior and characteristics of MSCs, further investigation is required on their exact roles and mechanisms. In addition, while performing cultures with serum-free media, studies should pay attention to the source or amounts of trace elements added to the MSC media.

## 8. Ascorbic Acid

Ascorbic acid acts as an antioxidant factor in cell cultures. Although it is not so commonly added in MSC media, studies have shown that it is involved in promoting MSC growth and proliferation when supplied to the culture medium. The study of Choi et al., revealed that the addition of L-ascorbate-2-phosphate in the culture medium enhanced the proliferation rate of human BM-MSCs in a dose-dependent manner [104]. Addition of 250 μM of L-ascorbate-2-phosphate induces the highest proliferation rates without any effect on cell morphology and MSC phenotype, while addition of 500 μM hinders the proliferation [104]. Same results using the same concentration of ascorbic acid supplementation were observed by other studies using human MSCs [83] from adipose tissue [105]. Another study using also human BM-MSCs showed similar results with the addition of L-ascorbate-2-phosphate up to 3 mM, but without differences between the concentrations tested [106]. In the same study, L-ascorbate-2-phosphate promoted MSC expansion from mononuclear cell population. These results support that ascorbic acid is a beneficial additive for MSC cultures.

## 9. Human Platelet Lysate

As mentioned earlier, culture medium enrichment by the addition of fetal bovine (fetal calf) serum (FBS/FCS) is still a common practice in many laboratories worldwide. It is rich in hormones, vitamins, transport proteins, trace elements, spreading, and growth factors [107,108]. In vivo, these are released upon blood coagulation and destined to help repair the site of injury, while in vitro, they promote cellular growth. The production raises both scientific as well as ethical concerns, in terms of xenogeneic compounds, reproducibility, and animal welfare, respectively. The whole blood needed is collected from unborn calves, which are a “by-product” of the meat producing unit. The use of fetal animals instead of adults is regarded to the low number of immunoglobulins in the calf serum. Collection is performed by harvesting the blood from the umbilical vein or more invasively directly from the heart by cardiac puncture. These procedures are performed right after the mother dams’ neck has been cut to ensure fetal death by anoxia and blood loss. To prevent suffering of the calf during the whole procedure, it has to be unconscious and must not breathe air at any stage, however, such safeguards are only recommendations and not legally defined [109]. After coagulation the liquid serum part is separated from the solid blood clot by centrifugation and can be further processed (filtration, quality control, packaging) for sale. When considering that in vitro cell culture is supposed to reduce the need for animals and the associated painful procedures, using animal derived compounds is contradistinctive. An estimation of the amount of FBS needed per anno leads to a calculated number of 1 million fetal calves necessary to meet the global requirements [109]. The fact that the main production countries are situated in regions with less legal restrictions concerning animal welfare, gives rise to even more ethical concerns regarding the use of FBS. Furthermore, the dependence from global meat markets and price variations, adds even more concerns [110,111]. 

On another aspect, addition of such ill-defined growth factor and protein cocktails and especially xenogeneic compounds is also not in the spirit of good manufacturing practice (GMP) guidelines and hinders transition of cell-based therapies to the clinics. In a desperate search for alternatives, various derivatives of human blood have been investigated [112]. Due to lower levels of growth factors and nutrients, plasma and serum prepared from anti-coagulated or coagulated whole human blood, respectively, are not widely used. However, human platelet lysate (hPL) has been shown to be an adequate substitute for FBS. This blood derivative can be manufactured from platelet concentrates by induction of bioactive molecule-release from the platelets. As platelet concentrates (PCs) are of human origin, already routinely collected by blood banks for the treatment of thrombocytopenia, and can be repurposed after a short shelf life of 5–7 days for research purposes [113,114,115,116,117,118], and its use is not accompanied by ethical or animal welfare concerns. Human platelet lysate has been used in cell culture already in the 1980s and by now has even proven to be superior to FBS at the same concentration in regard to promoting cell proliferation and maintaining MSC characteristics in several studies [113,114,115,116,117,119,120,121,122,123,124,125]. A summary from selected studies from 2005 to 2020 is listed in Table 2. Only studies that compared the performance to FBS as supplement were included. Used concentrations ranged from as low as 0.1% hPL supplementation to 10%, where in a majority of the trials, 5% hPL performed similar to 10% FBS. Stemness characteristics could be maintained or even improved, while one difference observed in some studies was a reversible change in morphology in media with one or the other supplement, with generally smaller cell sizes in hPL cultures. However, the mode of preparation may possibly influence the properties of the hPL. PCs can be prepared in 3 different ways: By the platelet rich plasma (PRP) method, from buffy coats, or by direct single donor platelet apheresis. For the PRP method whole blood is centrifuged to separate red blood cells from platelet rich plasma which is further centrifuged to reach the required concentration and material from 4–5 donors is pooled to reach the desired volume [126]. For the second method 4 buffy coat units from centrifuged whole blood are mixed with one plasma unit and centrifuged again, followed by separation and leukocyte depletion. The only method producing single donor PCs is by platelet apheresis. Optionally a pathogen inactivation or irradiation step is then performed. In order to produce hPL from the PCs there are again multiple options. A very common, efficient, and economical method is using single or multiple freeze/thaw cycles to lyse the platelets [113,116,117,118,120,123,124,125,127,128,129]. The concentrates are usually shock frozen at either −30 °C or −80 °C and reheated to 37 °C. Next to that, platelets can be activated by inducing thrombin generation, thus fibrinogen polymerization to fibrin and platelet degranulation. This can be achieved by the addition of calcium salts or one step further down the cascade, recombinant thrombin. Further methods are mechanical rupture by sonication, sometimes in combination with freeze thaw cycles or solvent/detergent treatment. With this in parallel to platelet activation, lipid enveloped viruses can be inactivated. Generally, to allow for more consistency multiple hPL units are again pooled (up to 109 units [130]). Lastly, any remaining fragments are removed, the pool is aliquoted and controlled before release. In recent years multiple commercial options have become available and some of them have already been under investigation in comparison to FBS (see Table 2) [131,132,133,134]. Suppliers include Sigma-Aldrich, Merck, PL Bioscience, Compass Biomedical and Stemcell Technologies. All of them offer their hPL in research grade, PL Bioscience additionally offers GMP grade and even pharmaceutical grade quality. For all hPL products in which fibrinogen is not depleted, heparin is needed to prevent medium gelation (coagulation) during culture, which can hamper cell proliferation and due to often porcine origin prevents the system from becoming fully humanized. To achieve this either recombinant human heparin or fibrinogen depleted hPL should be used in the future. In instances were only a small number of cells needs to be provided for a cell-based therapy (CBT), thus lower culture medium amounts are required autologous hPL can be used. Though in general, larger allogenic pooled hPL is regarded as the more promising alternative, due to higher availability. The downside of pooling is the increased risk of contamination with different pathogens.

All in all, hPL has proven to be equally suitable, if not an improvement over the classically supplemented FBS, represents an alternative, and its usage is one important step towards the establishment of xeno-free, humanized culture models. 

## 10. Serum-, Xeno-Free-, and Chemically Defined Media

Next to the switch from bovine or other animal-origin sera to human platelet lysate, other alternatives to fully humanize or potentially even develop chemically defined media are investigated for various reasons. Improving cell growth as well as phenotype is of great interest and specially to reduce the high variability encountered with non- or ill-defined medium components. To understand all the different approaches, firstly the various definitions used have to be examined. Chemically defined medium means that there is no supplementation with unknown composition, this includes blood derived supplements like serum, platelet lysates, but also plant hydrolysates. Highly purified components of different origin or recombinant products but with exact concentrations, can be added. As the origin can be from animals as well, chemically defined is not the same as animal-derived component free. Animal-derived component free (ADCF) stands for medium without supplements originating from animals, this usually includes humans as well. When no supplementation of serum is necessary the medium is called serum-free (SF), but may contain other protein fractions with plant or animal origin. As there could in both cases still be hydrolysates from bacteria or yeast for the ADCF media or other animal derived protein cocktails for the SF medium added, these terms not necessarily mean chemically defined. The term xeno-free is especially tricky as it depends on the origin of the to be cultured cell line. Xeno is of Greek origin and means “strange” and denotes compounds derived from a different species. Therefore, for human MSCs medium with human serum or platelet lysate is xeno-free, but the same media would not be if one would culture mouse MSCs in it [107]. Lastly, protein-free media are media that do not contain large proteins, but only small peptides. In this case, one can already see the limitations associated to these definitions. Where is the border to be set between protein and peptide? Further, recombinant human proteins can be produced in either bacterial or other mammal cell lines. This again raises question if they can be considered xeno-free and needs to be addressed in future media formulations [136].

Considering human cells for cell-based therapies, omitting xenogeneic compounds will rule out further immune reactions of the transplanted material and especially for bovine serum, and also the risk of transmitting prion diseases such as spongiform encephalopathy. Additionally, FBS production inevitably has high lot-to-lot variations, which requires pretesting before a new lot can be used for culture of MSCs [137,138]. With the transition to completely serum free chemically defined medium, further advantages arise. Blood-derived components, whether they are of animal or human origin, are heterogenous and, as already said, will inevitably have differences from batch to batch. This leads to less reproducible results and also causes a more heterogenous cell population [139]. With the use of serum-free medium (SFM), it can be specifically tailored for the needs of the MSC fraction, enhancing selection already during isolation. Enhanced consistency is also in the spirit of GMP regulation and thus the logical next step. There are already ready-to-use media specifically for MSCs available from different suppliers that are either serum-free, xeno-free, or both. To give a few examples, StemPro™ MSC SFM and StemPro™ MSC SFM XenoFree™ (Thermo Fisher Scientific, Waltham, MA, USA), StemXVico (R&D Systems, Minneapolis, MN, USA), PowerStem MSC1 (Pan Biotech, Aidenbach, Germany), MSC NutriStem^®^ XF (Biological Industries, Kibbutz Beit-Haemek, Israel), PRIME-XV MSC Expansion SFM (IrvineScientific, Santa Ana, CA, USA), MesenCult™-ACF Plus (Stem Cell Technologies, Vancouver, Canada), and MSCGM-CD (Lonza, Basel, Switzerland). Other options are SFM designed, intended for culture of embryonic or induced pluripotent stem cells (iPSCs) that are adapted to suit MSC as well, e.g., mTeSR (Stem Cell Technologies). The advantage of these commercial options is that they comply to GMP regulations and undergo strict testing by the supplier, and as they are premixed, handling times and material requirements are minimized on the user side. Further, a lot-to-lot variation of the individual components can also be averted. On the downside, however, the exact formulations are not disclosed by the companies, and thus adaption and optimization to the specific cell source or intended application is not possible, and in comparison to other media options they are costlier. A summary of tested media including the origin of the cells they were tested on is listed in Table 3. Interestingly, in a study by Al-Saqi from 2015, bone marrow derived cells did not perform well in Mesencult XF medium compared to MSCs derived from adipose tissues. This further indicates unique needs of different MSC populations. For the generation of chemically defined medium in-house all aspects regarding medium ingredients and concentrations above are of great importance to support MSC growth; optimization of serum albumin, fatty acids, trace minerals, glucose content, and combination of growth factors. 

Common supplements, next to what has been discussed before, include insulin, transferrin, and ethanolamine in combination with the trace element selenium (e.g., ITS, SITE). The role of insulin is to help cells use glucose and amino acids, transferrin serves as iron transport protein as free iron has toxic effects on cells [140] and ethanolamine is necessary for phospholipid synthesis [141]. Exemplary compositions from different disclosed serum free media include simpler compositions of only a few ingredients: IMDM supplemented with bFGF, human albumin (most abundant protein in vertebrate plasma, important for colloidal osmotic pressure and binding of ions, fatty acids, steroids and drugs [142]), hydrocortisone (steroid hormone, promotes cell growth [143]), and SITE [144]. A more complex chemically defined medium for hMSCs was patented already in 1995 by Marshak [145]: IMDM supplemented with serum albumin, lipoprotein, transferrin, insulin, MEM vitamins, MEM essential amino acids, MEM nonessential amino acids, sodium pyruvate, GlutaMAX-I supplement (substitution for L-glutamine, which is more stable in culture conditions [146]), folic acid (central role in one-carbon metabolism, important for growth, differentiation, and survival [147]), Ascorbic acid 2-phosphate, Biotin (essential vitamin, relevant for fatty acid synthesis, amino acid, and energy metabolism [148]), vitamin B12 mix, trace element mix, FeSO_4_, nucleoside mix, antibiotic/antimycotic, and either PDGF ββ homodimer or M 5-hydroxytryptamine (serotonin, micromolar amounts can increase cell growth [149]). Both chemically defined and xeno-free is the formulation of Wu et al. [150] (IMDM, l-glutamine, sodium bicarbonate, insulin, transferrin, serum albumin, β-mercaptoethanol (potent reducing agent, prevents toxic levels of oxygen radicals [151]), chemically defined lipid concentrate, MEM essential amino acids solution, MEM non-essential amino acid solution, Vitamins solution, trace elements solution, hydrocortisone, l-ascorbic acid-2-phosphate, fibronectin, progesterone (hormone, enhances immunomodulatory function of MSCs [152]), putrescine (non-protein nitrogen base, associated with proliferation in mammalian cells [153]), serotonin, epidermal growth factor, basic fibroblast growth factor, platelet-derived growth factor, insulin-like growth factor) [150]. The exact compositions of each of the three examples can be found in Table 4. Another option for labs is to get a custom-made medium from a commercial vendor. Such tailormade media can be procured from Cell Culture Technologies, a Swiss biotech company [154], or also larger biotech suppliers like Thermo Fisher Scientific, R&D Systems, and Biological Industries. Improvement on current animal component and undefined media compositions is the first step to improved cell culture concepts, further improvements could be made in investigation of culture media that are designed to specifically meet the needs of the cells in different “phases” of their life cycle, as current approaches do not consider such differences during culture.

Although the classical serum supplemented medium is still widely used and produces satisfying cell yields, transition away from animal-derived as well as poorly defined medium components is an essential step towards clinical relevance and further product development. Clearly, increased costs and challenges in the transition phase hinder a lot of research groups to switch to chemically defined medium. However, even small changes, one at a time, starting by omitting animal-derived products over to disposal of undefined sera and ultimately even proteins can improve the cultivation system and ensure more consistency.

## 11. Hypoxia

Thinking of parameters outside of the culture medium, itself long-overlooked, is the so called “normoxic” environment. When laboratories report culture of cells under normoxic conditions, they mean ambient oxygen concentrations of 21%, yet the question arises if this is really physiologically “normal” for the cultured cells. Some cell types in the human body are adapted to these levels, like cells of the respiratory system, skin, and the oral cavity. However, if we follow the dissolved oxygen within the blood stream to its destination in organs and tissues, a lot of lower concentrations are the norm than what is termed “normoxic”. Depending on the source tissue in vivo, oxygen levels between 18% (lungs) and as low as 1% (cartilage, bone marrow) are found [162]. Therefore, the actual physiological normoxic conditions for MSCs are actually rather low and are regarded as hypoxia. One must also take into account that atmospheric oxygen levels are not the same as the amount of oxygen dissolved in the medium. In case of a standard culture flask in a typical incubator, 20% oxygen in the surroundings means an estimated dissolved oxygen content of 100% in the medium. The amount of diffusion of oxygen into the culture medium is determined by physical laws influenced by a plethora of parameters. Ambient O_2_ concentration [137], O_2_ partial pressure determined by altitude, temperature, culture volume, and surface area have to be taken into account and monitored closely for studies on effect of oxygen levels on cells. With higher levels of dissolved oxygen other problem can emerge, the formation of so-called reactive oxygen species (ROS), for example peroxides, super-oxides, and hydroxyl radicals. Although they are a by-product in normal cell metabolism, dramatic increase of them can harm cellular structures, this is termed oxidative stress. Decrease of environmental O_2_ levels also reduces the formation of these ROS [163,164]. In the last years more and more studies have indicated, that mimicking the native niche conditions where the cells to be expanded reside in can improve their viability, promote proliferation, and maintain their in vivo properties. An indication of how important hypoxic conditions are in a cellular context is the large group of gene promoter regions that are responsive to hypoxia (hypoxia responsive elements HRE). The key player for activation is hypoxia inducible factor I (HIF1), which is a dimeric transcription factor. Expression of the two subunits is not oxygen dependent, but stability of the α-subunit is. Under normoxic conditions it is rapidly targeted for degradation, only in low oxygen concentration it is able to accumulate intracellularly and associate with the β-subunit and become functional [165]. In order to benefit from the positive effects of hypoxia in in vitro settings, such an environment has to be established first. Practical considerations of pros and cons of different methods shall be discussed. 

One of the easiest and simplest forms is the acquisition of a hypoxic sub-chamber (for example from BioSpherix [166] or Stemcell Technologies [167]), which is cost-saving and easy to handle. On the downside is of course the inevitable reoxygenation events every time a media change, microscopic evaluation, etc. is performed. Most models do not allow for tight oxygen control; thus, leakage and consistent low levels cannot be secured. For very low levels repeated re-flushing with nitrogen might be necessary to keep them. A bit more controlled is the use of a standard incubator with nitrogen gas supply, which is able to establish hypoxia by controlling oxygen levels next to humidity and temperature. However, handling with the culture vessels still requires removal from the hypoxic environment and especially for laboratories with multiple persons using the same hypoxia incubator, constant open and closing can lead to unwanted reoxygenation. The increase of size can be regarded as a two-sided coin; on one side, increased space allows for larger experiments and multi-person use, but on the other side, more gas is needed to establish the desired atmosphere as a sub-chamber does. Complete hypoxia workstations, optionally with gloves, circumvent any disruptions in oxygen levels, but are very costly and require a lot of attention for maintenance. They can be large enough to fit microscopes or other imaging systems within and allow for routine maintenance of cells like media exchanges or passaging in the desired environmental conditions [168]. Downsides to these workstations are that, on the other hand, access to the inside, for example for cleaning, is rather difficult. A different approach is to only mimic hypoxia by addition of Cobalt Chloride (CoCl_2_) or Deferoxamine Mesylate (DFO). These chelating agents can, after short hypoxic treatment, stabilize HIF at non-hypoxic conditions. This treatment has many limitations, cannot emulate real hypoxia, and has known and maybe even not yet discovered side effects on the cells, thus we will not go further into detail on this method.

Although a general improvement of cell yield under hypoxic conditions can be concluded (see Table 5) [61,113,169,170,171,172,173,174,175,176,177,178,179,180,181], some research groups could not confirm the beneficial effects of hypoxic culture conditions [182,183,184,185]. This might also be caused by high variations within the applied parameters. First and foremost is the inconsistent definition of hypoxia, tested O_2_ concentrations range from 1% to 5%, and secondly, fluctuations due to the aforementioned different methods of establishing hypoxic conditions in a laboratory setting are not taken into account. An important step towards harmonizing future results is the employment of non-invasive monitoring of the dissolved oxygen. Another point is the distinction between short term hypoxic treatments, or continuous culture under physiological O_2_ levels. Cells destined for transplantation are sometimes pre-conditioned for a few hours before administration, whereas other approaches try to keep cells already starting from the isolation procedure [186] until the transplantation at lowered oxygen concentrations. 

Nevertheless, a switch from the prevailing atmospheric oxygen levels during culture is an important advance in providing the cells a more physiological environment. 

## 12. Isolation and Passage Cell Seeding Densities

Studies on cell seeding density, although limited, indicate that it does have an effect on the proliferation rate and morphology of the cells. For primary MSCs, Sotiropoulou et al., found that seeding density of 1000 cells/cm^2^ of primary bone marrow mononuclear cells can result in significantly increased number of adherent MSCs after 1 week of culture compared to higher seeding densities tested, without any alterations on their phenotype, colony-forming, and immunosuppressive capacities [40]. Regarding passaged cells, studies have shown that seeding BM-MSCs in low densities (50–100 cells/cm^2^) increases their proliferation rates compared to higher densities (500–5000 cells/cm^2^) [40,187,188,189]. In addition, studies using extremely low seeding densities (1.5–10 cells/cm^2^) have showed that proliferation rates are inversely proportional to the seeding density of BM-MSCs, and that the cells maintain their immunophenotype, and morphological and self-renewal characteristics during culture [187,189,190,191]. Furthermore, differences in the seeding density of MSCs can also alter their gene expression patterns. In particular, studies have shown that human MSCs cultured in low seeding density (200 cells/cm^2^) can maintain their stem and immunomodulatory gene expression profiles better than when seeded in higher density (5000 cells/cm^2^) [192,193]. In general, seeding MSCs in low densities results in higher proliferation rates and better preservation of their physiological characteristics. This is also advantageous with regard to the limited number of primary MSCs that must be expanded after isolation from the tissue.

## 13. Cell Detaching Methods

During in vitro expansion, MSCs are usually cultured for multiple passages. It has been reported that human BM-MSCs are genetically stable and maintain their immunomodulatory and differentiation capacities up to passage 4 [194]. Therefore, in clinical applications, MSCs can be successfully used up to 4 passages. During passaging, efficient cell detachment is imperative in cultures of adherent cells like MSCs, without causing any damage. The most common and effective methods involve the use of enzymatic solutions to release the cells from the plastic culture surface by degrading their surface attachment proteins. Trypsin is a serine protease and is the most common enzymatic mean for dissociation of adherent cells. An alternative to Trypsin is Accutase, a proteolytic and collagenolytic enzymatic solution, which in comparison to Trypsin does not contain mammalian or bacterially derived proteins. Trypsin and Accutase have been reported to be the most suitable cell detaching reagents for human primary [195] and cultured MSCs [196,197,198] compared to other enzymatic (Collagenase, Prolyl-specific Peptidase) and non-enzymatic cell dissociation solutions (cell dissociation buffers, ethylenediamine tetra-acetic acid (EDTA)) and cell scraping, with significantly lower incubation times, increased viability, and higher yields of detached cells. Although different types of culture surface coatings do not significantly influence the effectiveness of enzymatic dissociation solutions, the results from the study of Salzig et al., suggest that the efficiency of different enzymatic detachment methods and corresponding cell viability following detachment are significantly affected by the culture media used [198]. However, due to its proteolytic activity, it has been reported that trypsin can significantly reduce the expression of MSC surface markers [195,198] and change the expression of several non-coding RNAs, resulting in several transcriptomic changes [199]. Although some non-enzymatic buffers seem to minimize the adverse effects of other methods on the detached MSCs [195,198,200], most studies suggest that due to the long incubation times required for non-enzymatic detachment means, Trypsin is comparatively a more beneficial method for quick preparation of MSC suspensions [195,196,200].

Although trypsinization is the most common laboratory method used for detaching MSCs, in order to avoid the negative impact of proteolytic degradation on cell membrane during enzymatic dissociation, new techniques are developed, for example thermoresponsive detachment of adherent cells. Adherent MSCs can be detached from the culture surface by changing the temperature. However, without appropriate culture substrate, MSCs must be exposed to very low/non-physiologic temperatures to facilitate detachment [201]. For this reason, a thermoresponsive polymeric coating, usually poly(N-isopropylacrylamide), is applied on the cell culture surface. These thermoresponsive surfaces have been reported to promote human MSC adhesion, colony formation and proliferation [202,203], and enable their detachment with temperature reduction below a critical solution temperature [202,203,204], without any changes on the MSC specific marker profile and morphology [205,206]. A comparative study by Yang et al., suggests that thermoresponsive detachment of MSC from poly(N-isopropylacrylamide) films is as effective a method as trypsinization [206]. These cell detachment methods are promising in avoiding the proteolytic adverse effects of trypsin, although they are still not so well optimized as routine methods such as trypsinization.

Other cell dissociation methods have been recently developed, using for example acoustic pressure [207], light-induced plasmonic substrates [208], and non-enzymatic arginine-based solutions [209] to detach adherent cells. These techniques show very promising results, but their application on MSCs has not been investigated yet.

## 14. Conclusions

Human MSCs are considered one of the most prominent types of cells for cellular therapies, due to their multipotent and regenerative capacities. However, due to their limited number after isolation, in vitro expansion is required to achieve a sufficient number of cells for clinical and research applications. This expansion step is usually performed in a standard 2D culture setup that requires a lower number of seeded cells due to its robustness and simplicity compared to more recently developed advanced 3D culture methods. Therefore, in order to improve the relevance of in vitro applications, it is imperative to preserve the physiological characteristics of MSCs during this necessary in vitro expansion step.

**Figure 1:**
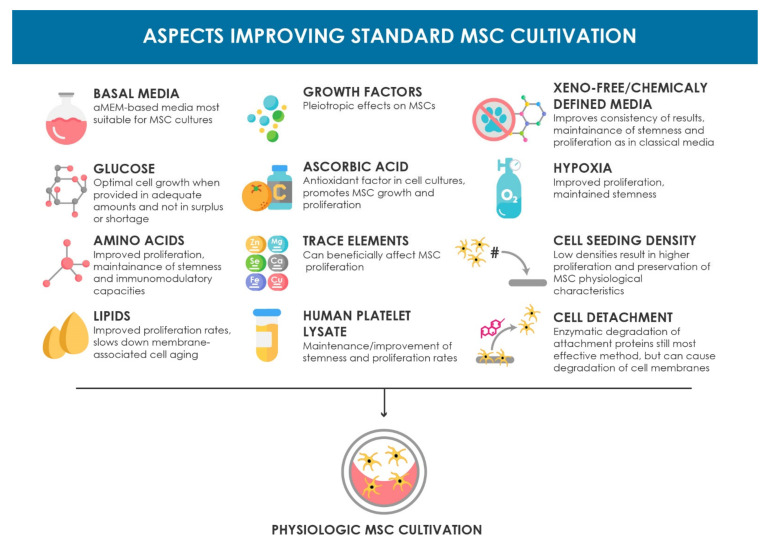
Schematic overview of the aspects improving the standard MSC cultivation setup discussed in this review. The beneficial biological effects are summarized for each topic.

Many optimization approaches for in vitro expansion of human MSCs focus on well-formulated and chemically defined culture media. An overview of the major components of culture media and additional optimization approaches and their biological effects mentioned in this review are summarized in Figure 1. However, apart from the components mentioned there, there are many more included in their formulations that can potentially affect cell behavior and were not given enough attention so far, and therefore require focused research. Furthermore, apart from their chemical properties, alterations in the physical properties of the media like osmolarity are often neglected. Similarly, the influences of exposure to light and temperature on the characteristics and behavior of the cells during culture are widely ignored and present a promising aspect for further investigation [210,211,212,213].

In order to maximize the benefits of these aspects on MSC culture, it is important that each of these elements is thoroughly investigated, so their exact underlining mechanisms and concomitant effects on the cells are identified. This can also expand the range and validity of analytics for assessing MSC expansion cultures. Many of the studies in this review evaluated the effect of different aspects on expanding MSCs by mainly measuring total number of cells, doubling times or time to reach confluency. Although these methods may give representative indications of the effects on cultured MSCs, due to the different metric approaches, comparison and relevance between the results are not always accurate. In addition, these methods only cover cell attributes like proliferation and growth rates. Thereby, possible differences in a more thorough biological level, like gene expression, or surface marker and proteomic alterations, are often disregarded. Attempts to identify alterations on the cells immunomodulatory profile by prolonged ex vivo cultivation have been made in “standard” cultivation settings [214], as understanding the cellular response to expansion can influence the therapeutic potential of a CBT. Optimization approaches can also greatly influence these cell properties, which can be even more relevant for treatment success than solely greater cell numbers. For that reason, the introduction of specific criteria and analytics for the evaluation of the effects of different aspects during in vitro expansion of human MSCs, similar for example to the ones the ISCT established for defining MSCs, would be beneficial for the significance and relevance of future studies in this research topic.

While most of the elements mentioned in this review have been proven to be beneficial for human MSC in vitro cultures when examined solely, the impact of the combination of different elements should also be investigated. Most papers studied only one parameter, and only rarely were two investigated. For future media composition optimization, multiparametric analysis with carefully considered design of experiments should reveal more information on the interplay of different components. Furthermore, MSCs inherently present high donor and source variability in their characteristics and properties. For that reason, some of the studies included in this review have reported different effects of the same investigated in vitro culture parameter on different types of MSCs. Therefore, further research should be directed towards formulation of MSC type-specific culture media and protocols to avoid variability in preserving the physiological status of cultured MSCs.

In conclusion, the approaches discussed in this review constitute the basic principles for MSC culture and seem to be advantageous for the preservation of physiological MSC characteristics during in vitro expansion. To fully recapitulate the in vivo conditions, further strategies such as 3D passage free culture will have to be developed further. The purpose of this review is to provide the readers with a broad variety of up-to-date approaches on standard expansion of human MSCs, that can help them optimize their current culture protocols and improve the efficacy and physiological relevance of their MSC cultures, as well as serve as a baseline for additional investigation and further optimization of standard cultivation of MSCs in vitro.

## Figures and Tables

**Table 1:** Different types of basal media.

Media Name	Description
DMEM	Dulbecco’s modified Eagle’s medium (MEM)
DMEM/LG/L-G	Dulbecco’s MEM (DMEM) with 1000 mg/mL glucose and L-glutamine
DMEM/HG/L-G	DMEM with 4500 mg/mL glucose and L-glutamine
DMEM/HG/GL	DMEM with 4500 mg/mL glucose and Glutamax
IMDM	Iscove’s modified Dulbecco’s medium with L-glutamine
aMEM	MEM alpha
aMEM/L-G	MEM alpha with L-glutamine
aMEM/GL	MEM alpha with Glutamax

**Table 2:** Human platelet lysate (hPL) as alternative to bovine serum; selected studies from 2005 to 2020 including commercially available products. Information is provided on starting material, preparation technique and culture conditions.

Starting Material	Solution	Pooled (PC)	Platelet Counts (×10^9^/mL)	Platelet Lysis		Expanded Cells	Supplementation	Outperformed FBS?	Ref.
				(Freeze/Thaw)	(Others)				
Aph-PC	Plasma	10	1	−80 °C	-	BM-MSC	5%	yes	[120]
BC-PC	Plasma	10–13	0.95	−30 °C	-	UCB-MSC	10%	yes	[124]
Exp Aph-PC	Plasma	yes	-	−80 °C	-	BM-MSC	10%	yes	[113]
Exp Aph-PC	Plasma	-	-	−80 °C	-	BM-MSC	5%	no	[118]
Aph-PC	Plasma	-	-	−80 °C	-	BM-MSC	8%	no	[129]
Aph-PC	Plasma	-	1.0–1.3	-	S/D	AD-MSC	10%	no	[135]
Aph-PC	Plasma	10	-	−80 °C	-	BM-MSC, UCB-MSC	5–10%	yes	[128]
BC-PC, leuko depleted	Plasma	-	-	−80 °C	-	AD-SC	5%	yes	[123]
Aph-PC	Plasma	4–6 don	-	−80 °C	Sonication	BM-MSC	10%	yes	[119]
BC-PC	Plasma	2	-	−30 °C	-	BM-MSC, AD-MSC	2.5–10%	yes	[121]
BC-PC	Plasma	2	-	−30 °C	Thrombin	BM-MSC, AD-MSC	2.5–10%	yes	[121]
BC-PC	Saline	6	3.34	−80 °C	-	AD-MSC	5%	yes	[125]
BC-PC	Plasma	6	3.58	−80 °C	-	AD-MSC	5%	yes	[125]
Exp Aph-PC	Plasma	5 don	1	−80 °C	CaCl_2_	BM-MSC	10%	yes	[114]
Exp BC-PC	Plasma	yes	-	−80 °C	-	BM-MSC	10%	yes	[117]
BC-PC, path. Inactivated	Add Sol	12 don	1	−80 °C	-	BM-MSC	10%	yes	[127]
BC-PRP	Plasma	10–20	10	−196 °C	Lyophilization, Irradiation	BM-MSC	5%	yes	[122]
PL-Serum	-	49–109	-	-	CaCl_2_ 20% *w*/*v*	commercial BM-MSC	10%	yes	[130]
BC-PC	Plasma	2	2.03	−25 °C	-	AD-MSC	1–10%	yes	[116]
BC-PC	TSOL	2	0.91	−25 °C	-	AD-MSC	1–10%	yes	[116]
Exp BC-PC	Plasma	2	0.41	−25 °C	-	AD-MSC	1–10%	yes	[116]
Exp BC-PC	TSOL	2	0.19	−25 °C	-	AD-MSC	1–10%	yes	[116]
Exp Aph-PC	-	3–4 don	-	−80 °C	-	AD-MSC	0.1–1%	yes	[115]
**Brand Name**	**Supplier**	**Pooled (PC)**	**Platelet Counts (×10^9^/mL)**	**Platelet Lysis**		**Expanded Cells**	**Supplementation**	**Outperformed FBS?**	**REF**
				**(freeze/thaw)**	**(others)**				
PLTMAX	Sigma Aldrich	yes	-	-	-	AD-MSC	5%	yes	[134]
MesenCult hPL media	Stemcell Technologies	yes	-	-	-	AD-MSC	10%	yes	[134]
PLUS™ hPL	Compass Biomedical	yes	-	-	-	BM-MSC	5%	yes	[131]
PLTmax	MERCK	yes	-	-	-	AD-MSC	1–10%	yes	[132]
phPL	PL BioScience	yes	-		-	BM-MSC	10%	yes	[133]

(Abbreviations: AD—adipose tissue derived, add sol—additive solution, Aph—apheresis, BC—buffy coat, BM—bone marrow derived, don—donor, Exp—expired, FBS—fetal bovine serum, MSC -mesenchymal stem cell, PC—platelet concentrate, PL—platelet lysate, PRP—platelet rich plasma, S/D—solvent/detergent, UC—umbilical cord derived UCB—umbilical cord blood derived).

**Table 3:** Commercially available, chemically defined/serum-free/xeno-free media tested for expansion of hMSCs; including selected studies from 2010–2021. Information is given on brand name, supplier, any added supplements, the cell source, cultivation parameters, and stem cell characteristics.

Basal Medium	Supplier	Add. Supplements	Cells Expanded	Cultivation Time	Outperformed CTL Medium?	Diff Capacity	Cell Surface Markers	Ref.
MSCGM-CD	Lonza	-	UC-MSC	5–7 passages	n.t.	unaltered	unaltered	[38]
MesenCult	Stemcell Technologies	-	hESC-derived MSCs	-	yes	n.t.	unaltered	[155]
Mesencult-XF™	Stemcell Technologies	-	AD-MSC, BM-MSC	-	yes	improved (AD-MSC), decreased (BM-MSC)	altered (BM-MSC)	[156]
StemPro MSC SFM XenoFree™	Life Technologies	-	BM-MSC	up to p4	no	n.t.	n.t.	[139]
Mesencult-XF™	Stemcell Technologies	-	BM-MSC	up to p4	no	unaltered	unaltered	[139]
BD Mosaic™ Mesenchymal Stem Cell Serum-Free media	BD Biosciences	-	BM-MSC	up to p4	no	unaltered	unaltered	[139]
StemPro^®^ MSC SFM XenoFree, Invitrogen	Life Technologies	-	AD-MSC, BM-MSC	-	yes	altered	unaltered	[157]
StemPro MSC SFM XenoFree™	Life Technologies	PDGF-BB, bFGF, TGF-β1	ASC line, BM-MSC	up to p9	yes	unaltered	unaltered	[158]
StemPro^®^ MSC SFM	Life Technologies	PDGF-BB, bFGF, TGF-β1	BM-MSC	8 passages	no	unaltered	unaltered	[159]
StemPro MSC SFM Xenofree	Life Technologies	-	BM-MSC, UC-MSC, AD-MSC	7 days	yes	n.t.	n.t.	[160]
MSC Nutristem XF	Biological Industries	-	BM-MSC, UC-MSC, AD-MSC	7 days	yes	n.t.	n.t.	[160]
MesenCult-XF	Stemcell Technologies	-	BM-MSC, UC-MSC, AD-MSC	7 days	yes	n.t.	n.t.	[160]
StemXVivo SFM Human MSC Expansion Medium	R&D Systems	-	BM-MSC, UC-MSC, AD-MSC	7 days	yes	n.t.	n.t.	[160]
RoosterNourish-MSC XF	RoosterBio, Inc.		BM-MSC	up to p5	no	unaltered	unaltered	[161]
StemMACS-MSC Expansion Media Kit XF	Miltenyi Biotec		BM-MSC	up to p5	no	unaltered	unaltered	[161]
MSC NutriStem XF	Biological Industries		BM-MSC	up to p5	no	unaltered	unaltered	[161]
StemXVivo SFM Human MSC Expansion Medium	R&D Systems		BM-MSC	up to p5	no	unaltered	unaltered	[161]

(Abbreviations: AD—adipose tissue derived, Add.—additional, BM—bone marrow derived, CD—chemically defined, CTL—control, diff—differentiation, hPL - human platelet lysate, MSC—mesenchymal stem cell, n.t.—not tested, p—passage, SFM—serum free medium, UC—umbilical cord derived, XF—xeno-free).

**Table 4:** Composition of three disclosed chemically defined cell culture media for MSCs.

**Ref.**	[144]	[145]	[150]
**Basal Media**	IMDM	IMDM	17.7 g/L IDMD
**Supplemented With**	17.91 ng bovine FGF/mL	5 mg/mL human serum albumin	5 mM l-glutamine
	2.80 mg/mL human albumin	100 μg/mL human Ex-Cyte lipoprotein	3.024 g/L sodium bicarbonate
	27. 65 µM hydrocortisone	2 μg/mL saturated human transferrin	10 mg/L rh insulin
	1.18% SITE (S4920; containing 0.5 µg/mL sodium selenite, 1.0 mg/mL bovine insulin, 0.55 mg/mL human transferrin, 0.2 mg/mL ethanolamine; 100-fold concentrate)	10 μg/mL rh insulin	10 mg/L rh transferrin
		1.0% 100 × MEM vitamins	4 g/L rh serum albumin
		0.89% MEM essential amino acids	55 μM β-mercaptoethanol
		0.4% MEM nonessential amino acids	0.1% chemically defined lipid concentrate
		1 mM sodium pyruvate	2% MEM essential amino acids solution
		1 mM GlutaMAX-I supplement	1% MEM non-essential amino acid solution
		10 μg/mL folic acid	1% Vitamins solution
		10 μM ascorbic acid 2-phosphate	0.1% trace elements solution
		1.0 μg/mL Biotin	50 μg/L hydrocortisone
		1.36 μg/mL vitamin B12 mix	50 mg/L l-ascorbic acid-2-phosphate
		500fold diluted trace element mix	5 mg/L rh fibronectin
		4 × 10^−8^ M FeSO_4_	5 μg/L progesterone
		10 μg/mL nucleoside mix (ribonucleosides, 2′-deoxyribo-nucleosides, uridine, and thymidine)	10 mg/L putrescine
			2 mg/L serotonin
			10 ng/mL rh EGF
		1.0% antibiotic/antimycotic	10 ng/mL rh basic FGF
		10–20 ng/mL rh PDGF ββ homodimer or 10^−5^ to 10^−6^ M 5-hydroxytryptamine	10 ng/mL rh PDGF
			10 ng/mL rh IGF

(Abbreviations: EGF—epidermal growth factor, FGF—fibroblast growth factor, rh—recombinant human, IGF—insulin-like growth factor, IMDM—Iscove’s modified Dulbecco’s medium, MEM—minimum essential media, PDGF—platelet-derived growth factor).

**Table 5:** Hypoxic conditions for expansion of hMSC; selected studies from 2007–2020. Information is given on the cell source, exposure to hypoxic conditions and on stem cell characteristics.

Cells	O_2_ (%)	Exposure Time	Outperformed 21% O_2_?	Diff Capacity	Cell Surface Markers	Other Cultivation Parameters Analyzed	Ref.
BM-MSC	2	6 weeks	yes	unaltered	n.t.		[169]
BM-MSC	1–3	16 h	no	n.t.	n.t.	HGF stimulation	[184]
BM-MSC	5	up to p4	yes	unaltered	unaltered	hPL	[113]
BM-MSC	2	14 d	yes	n.t.	unaltered		[113]
UC-MSC	1.5–5	3 d	yes	n.t.	n.t.		[171]
BM-MSC	1–5	14 d	no	decreased	unaltered		[182]
BM-MSC	1	84 days	yes	improved	unaltered		[172]
BM-MSC	5	up to p3	yes	improved	unaltered		[173]
AD-MSC	2	7 d	yes	improved	unaltered		[174]
BM-MSC	1	up to 90 d	yes	improved	upregulated		[175]
BM-MSC, AD-MSC, AF-MSC, UCB-MSC	1	7 d	depending on cell source (prenatal yes, postnatal no)	n.t.	n.t.	prenatal + postnatal material	[185]
AD-MSC	2	up to 21 d	yes	unaltered	unaltered		[176]
UCB-MSC	5	5 d	yes	n.t.	unaltered		[179]
AD-MSC	5	up to 14 d	yes	n.t.	slightly altered	lean + obese donors	[178]
BM-MSC	5	up to p15	yes	unaltered	n.t.	donor age	[177]
BM-MSC	1–4	up to p2	no	unaltered	unaltered		[183]
UCB-MSC	3	5 d	yes	unaltered	unaltered	Ca^2+^	[61]
AD-MSC	5	up to p28	yes (until passage 23)	n.t.	n.t.		[180]
AD-MSC	1	48 h	yes	decreased (osteogenic) increased (chondrogenic)	unaltered		[181]

(Abbreviations: AD—adipose tissue derived, AF—amniotic fluid BM—bone marrow derived, diff—differentiation, HGF—human growth factors, hPL—human platelet lysate, MSC -mesenchymal stem cell, n.t.—not tested, p—passage, UC—umbilical cord derived, UCB—umbilical cord blood derived).

## Data Availability

Not applicable.

## Abbreviations

2D	two-dimensional
3D	three-dimensional
ADCF	animal-derived component free
BM-MSCs	bone marrow-mesenchymal stem cells
EAAs	essential amino acids
EGF	epidermal growth factor
FBS	fetal bovine serum
FGF-2 and -4	fibroblast growth factor-2 and -4
GMP	good manufacturing practice
HB-EGF	heparin-Binding Epidermal Growth Factor
hPL	human platelet lysate
HIF1	hypoxia inducible factor I
IGF	insulin-like growth factor
ISCT	International Society for Cellular Therapy
MSC(s)	mesenchymal stem cell(s)
NEAAs	non-essential amino-acids
PDGF-BB	platelet derived growth factor-BB
PCs	platelet concentrates
ROS	reactive oxygen species
PRP	platelet rich plasma
SF	serum-free
VEGF	endothelial growth factor
